# Urothelial Carcinoma of the Urinary Bladder in Young Adults: Presentation, Clinical behavior and Outcome

**DOI:** 10.1155/2011/480738

**Published:** 2011-11-22

**Authors:** Michael Nomikos, Athanasios Pappas, Maria-Emmanouela Kopaka, Stavros Tzoulakis, Ioannis Volonakis, Georgios Stavrakakis, Georgios Avgenakis, Ploutarchos Anezinis

**Affiliations:** ^1^Urology Department, Venizelion General Hospital, 71400 Heraklion, Crete, Greece; ^2^Department of Pathology, Evagelismos General Hospital, 11527 Athens, Greece

## Abstract

*Introduction.* There is not much evidence regarding clinical behavior of bladder cancer in younger patients. We evaluated clinical characteristics, tumor recurrence and progression in patients younger than 40 years old with urothelial bladder carcinoma. *Methods.* We retrospectively reviewed the medical records of 31 patients less than 40 years old who were firstly managed with bladder urothelial carcinoma in our department. Data were analysed with the Chi-square test. *Results.* Mean age was 31.7 years. Mean followup was 38.52 months (11–72 months). Nineteen (61%) patients were diagnosed with GII and 2 (6%) patients with GIII disease. Five (16%) patients presented with T1 disease. Three (9%) patients with invasive disease underwent cystectomy and adjuvant chemotherapy and one developed metastatic disease. Ten (32%) patients recurred during followup with a disease free recurrence rate of 65% the first 2 years after surgery. From those, 1 patient progressed to higher stage and three to higher grade disease. No patient died during followup. *Conclusions.* Bladder urothelial carcinoma in patients younger than 40 years is usually low stage and low grade. Management of these patients should be according to clinical characteristics and no different from older patients with the same disease.

## 1. Introduction

Bladder cancer is a disease that afflicts mostly the middle-aged or the elderly people, as the majority of any other cancer type. The median age of diagnosis of bladder urothelial carcinoma is 69 years in males and 71 years in females, but the disease can occur at any age, even in children [1, 2]. Studies provide evidence that p53 gene product overexpression is common in bladder cancer in young patients [3]. There is still conflicting evidence regarding clinical behavior, disease recurrence, and progression in young patients who have been diagnosed with urothelial bladder carcinoma. The primary aim of this study is to evaluate clinical characteristics, disease recurrence, and progression in patients up to 40 years of age who have been diagnosed and treated with urothelial bladder cancer in our department.

## 2. Patients and Methods

We retrospectively reviewed the medical records of 31 consecutive patients aged 40 years or younger with bladder urothelial carcinoma, which have been initially diagnosed and treated in our department. Data were recorded regarding their presenting symptoms, family history, and any exposure to occupational risk factors. The time from the onset of symptoms until the diagnosis was established, and initial transurethral resection pathology, tumor diameter and location, mode of intravesical instillation therapy, number of recurrences, time till first recurrence, and disease progression to different stage or grade were also collected and recorded. Staging and grading of bladder cancer was evaluated according to the tumor node metastasis classification (TNM) and the World Health Organization (WHO) [4, 5]. The mean follow-up period was 38,52 months (11 to 72 months). Data were analyzed with the Chi-square test. A *P* Value of less than 0.05 was considered statistically significant. Data were analyzed using SPSS version 13.

## 3. Results

Between February, 2003 and January, 2010, thirty-one patients (19 males and 12 females) with a mean age at the time of diagnosis of 31.7 years (21–39 years) were firstly diagnosed with bladder urothelial carcinoma in our department. In our series, the male-to-female ratio was 1,6 : 1. Fourteen patients (45,16%) were under the age of 30, and seventeen (54,83%) patients were between 31 and 40 years old. Macroscopic hematuria was the presenting symptom in 25 (80.6%) patients. Twenty seven (87%) of our patients were cigarette smokers, with a mean number of 411 packs per year, which shows a strong relation between tobacco smoking and bladder cancer. Cigarette smoking is a known prominent risk factor, which triples the risk of developing bladder cancer [6–8]. The clinical characteristics of young patients with bladder cancer are presented in Table 1. The mean time from the onset of symptoms until diagnosis was established was 78,64 days (range 9–134 days). Ultrasound of the urinary tract was diagnostic in all cases. Cystoscopy, also, confirmed the ultrasound findings in all cases. Four of them (13%) had a family history of bladder cancer, and one of them had a family history of kidney cancer. Regarding exposure to occupational risk factors, three (9,6%) patients were working as plumber, motor mechanic, and car body repairer/painters. One female patient who presented with invasive bladder cancer had neurogenic lower urinary tract dysfunction managed with intermittent self-catheterizations and was suffering from recurrent urinary tract infections. All of them underwent transurethral resection of bladder tumor with additional biopsies from the tumor base. Final pathology revealed 28 (90%) patients with superficial urothelial bladder cancer and 3 (10%) patients with invasive disease. The overall mean tumor volume was 4,65 cm³ (1,1–16,8 cm³), and the mean tumor diameter was 1.4 cm (0.6–5.2 cm). Totally, 10 (32,2%) patients presented with multiple tumors (>2 tumors) at the time of diagnosis. The majority (58%) of the tumors were located in bladder trigone. Twelve (42%) patients with superficial bladder cancer had postoperative intravesical instillation therapy. Seven patients received epirubicin and 5 patients with high-grade disease received adjuvant BCG therapy. Overall, ten (32%) patients recurred during followup with a 65% disease free recurrence rate the first 2 years after surgery. Seven (25%) patients had 1 recurrence, two (7%) patients had 2 recurrences, and one patient had 3 recurrences. In the first year, recurrences were four times more in patients over 30 years old. Three patients progressed to higher grade (grade II to grade III) and one to higher to higher stage (Ta to T1). All patients (2 males and 1 female) with invasive bladder cancer underwent radical cystectomy (2 received ileal conduit, and 1 had Studer pouch). Two of them received adjuvant cisplatin-based chemotherapy. The patient with T3 disease developed metastasis to the lungs. None of the patients died during followup.

## 4. Discussion

Urothelial carcinoma of the urinary bladder is rare in young adults, as less than 1% of such tumors present in the first 4 decades of life [9]. Although many reports exist regarding clinical characteristics and treatment for such tumors, there is still much debate regarding clinical progression and prognosis. Indeed, some groups observed similar patterns of clinical behavior and prognosis for bladder cancer in young and older patients [10], whereas other investigators reported lower rates of recurrence and progression and better survival in younger patients [11–13]. Comparative data of the larger series in the literature of young adults with bladder cancer, regarding recurrence and progression, are presented in Table 2. In our study, 50% of patients under 30 years old presented with grade II disease, while 70% of patients over 30 years old had grade II disease. Furthermore, in the younger group, four patients (28%) had a recurrence but only one progressed to higher stage and one to higher grade. In the older group, six patients (35%) had a recurrence, but only two of them progressed to higher grade disease. In our study, none of the patients under 30 years of age presented with invasive bladder cancer, as opposed to the older age group, which included 3 patients (9%) with invasive disease. 

Our findings add to the growing evidence of the literature that patients under 40 years old usually present with low-stage and low-grade bladder cancer. Clinical behavior of high stage and high-grade disease seem to be similar to older people. Those patients should be managed aggressively especially when unfavorable prognostic factors coexist such as multifocality, high grade and stage, and tumors >3 cm [14–16].

An important finding of our study was the significant delay of approximately 80 days from the onset of symptoms till diagnosis was established with an ultrasound. This seems to be common in younger patients due to low incidence of bladder cancer in this age group and the predominance of benign causes of hematuria in this age group causing hesitancy for an aggressive work-up. 

In a recent meta-analysis, Paner et al. concluded that tumor recurrence and progression were infrequent in the first 2 decades and increased gradually in each successive decade, likely influenced by the increased proportion of higher-grade and higher-stage tumors [19]. In the presence of gross hematuria, a urinary tract ultrasound is the first work-up for establishing the diagnosis of bladder tumor. Although it cannot replace cystoscopy, it is a reliable diagnostic tool for the detection of bladder tumor especially for those young patients who hesitate to undergo a diagnostic cystoscopy. This observation was confirmed in our study, since all patients with an ultrasound finding of a bladder lesion were found to have bladder tumor in cystoscopy. 

Of course, there are possible limitations in our study. This is a retrospective audit with small number of patients and relatively sort followup, although similar to prior reports, making safe conclusions difficult to extract. Further larger well-conducted prospective studies with longer followup are needed to confirm long-term progression and outcomes especially in young adults with invasive urothelial bladder cancer. 

In children, adolescents, and young adults less than 20 years old, bladder TCC has a very low recurrence rate with excellent prognosis [20]. These findings should not be extrapolated to describe clinical behaviour of bladder TCC in patients between 30 to 40 years old who usually present with higher grade and stage and tend to recur more often [21, 22].

## 5. Conclusion

Younger patients with bladder cancer appear to have a more favorable prognosis, because they usually present with superficial stage and low-grade tumors. However, the risk of disease progression is the same, influenced by grade and stage at the time of presentation. Patients younger than 40 years old diagnosed with bladder cancer should be offered the same stage and grade appropriate management as older ones.

## Figures and Tables

**Table 1:** Presentation, clinical characteristics, and treatment modalities of young patients with bladder cancer.

Clinical characteristics of patients <40 years old treated for bladder cancer				
	Age (yr)			
	≤30	31–40	Total	P*
Patients, *n* (%)	14 (45.16)	17 (54,83)	31 (100)	
Mean age (range), yr	26,14 (21–30)	37,29 (31–39)		
Males/females	9/5	10/7	19/12	*P* = 0.46
Clinical characteristics at presentation				
(i) Macroscopic hematuria	10	15	25	
(ii) UTIs	1	1	2	
(iii) Microscopic hematuria	3	1	4	
Mean followup (range), mo	39,36	37,68	38,52	
Multifocality of bladder tumors (>2 lesions)	4	6	10	*P* = 0.39
Stage at presentation, *n*				
(i) Ta	10	13	23	
(ii) T1	4	1	5	
(iii) T2	0	2	2	
(iv) T3	0	1	1	
(v) T4	0	0	0	
Grade at presentation, *n*				
(i) Low (GI-II)	14	15	29	
(ii) High (GIII)	0	2	2	
Recurrence rate (%)	4 (28)	6 (35)	10 (32)	*P* = 0.28
Intravesical therapy of superficial cancer *n* (%)	7/14	5/14	12/28	
Cystectomy, *n* (%)	0	3	3	
Adjuvant chemotherapy for invasive cancer, *n* (%)	0	2	2	

**Table 2:** Comparative data of young patients with bladder cancer from other series.

Authors	Number of patients	Median followup (months)	Superficial bladder cancer (%)	Invasive bladder cancer (%)	Recurrence rates (%)	Progression to invasive bladder cancer (%)
Wen et al. [9]	30	72,8	76,6	23,4	50	8,3
Yossepowitch and Dalbagni [22]	74	28,1	83,4	16,6	38,7	16
Erőzenci et al. [18]	156	87	89,1	10,9	48,7	22,8
Perez et al. [17]	30	66	67,6	23,3	32	0,1
